# Cost Reduction Behaviors and Cost-Related Medication Nonadherence in Older Adults with Atrial Fibrillation

**DOI:** 10.1093/geroni/igab046.2342

**Published:** 2021-12-17

**Authors:** Benita Bamgbade, David McManus, Becky Briesacher, Darleen Lessard, Jordy Mehawej, Mayra Tisminestzky, Jerry Gurwitz, Jane Saczynski

**Affiliations:** 1 Northeastern University, Boston, Massachusetts, United States; 2 University of Massachusetts Medical School, Worcester, Massachusetts, United States

## Abstract

While factors such as forgetfulness may result in medication nonadherence, 2.7 million older adults in the US experience cost-related nonadherence (CRN). Limited research has explored CRN and associated cost-reduction behaviors (CRB) in older adults with atrial fibrillation. The objectives of this study were to 1) describe the prevalence of CRN, CRB and spending less on basic needs to afford medication and 2) examine factors associated with CRB among older adults with atrial fibrillation. Data were drawn from the Systematic Assessment of Geriatric Elements in Atrial Fibrillation (SAGE-AF), a prospective cohort of older adults with atrial fibrillation (>65 years). Using a self-administered survey, all participants completed a validated CRN measure. Chi-square and t-tests were used to evaluate differences in participant characteristics across CRB and significant characteristics (p<0.05) were entered into a logistic regression model. Participants (N=1244) were on average 76 years and 49% were female. Among all participants, 4.2% reported CRN; 69.1% reported CRB; and 5.9% reported spending less on basic needs. Compared to participants who did not engage in CRB, participants who engaged in CRB were less likely to be cognitively impaired and more likely to be a race/ethnicity other than non-Hispanic white; have Medicare insurance; and have comorbidities. CRB were common among older adults with atrial fibrillation and was associated with in-tact cognitive function, the presence of medical comorbidities and non-White race. Clinicians might consider providing patients with cognitive impairment additional support such as patient assistance programs or referrals to pharmacists for medication therapy management to assist with CRB.

